# RUMINATION MEDIATES THE ASSOCIATION BETWEEN REGIONAL GRAY MATTER VOLUME AND SUBTHRESHOLD DEPRESSION IN OLDER WOMEN

**DOI:** 10.1093/geroni/igae098.2678

**Published:** 2024-12-31

**Authors:** Dongmei Wu

**Affiliations:** The Clinical Hospital of Chengdu Brain Science Institute, MOE Key Lab for Neuroinformation, University of Electronic Science and Technology of China, Chengdu, Sichuan, China (People’s Republic)

## Abstract

This study aimed to identify distinctive characteristics of gray matter volume alterations in older women with subthreshold depression and to explore the mediating role of rumination in the relationship between changes in brain structure and depressive symptoms. A total of 102 older women residing in the community were divided into two groups: those with depression symptoms (DS) (n=50) and a healthy control (HC) group (n=52). Whole-brain two-sample t-tests on voxelwise gray matter volumes in the brains were conducted to identify gray matter volume alterations in older women with subthreshold depression, and mediation analysis was performed to test whether rumination mediates the impact of gray matter volumes on depressive symptoms in older women. Even after controlling for age, education level, monthly income, Mini-Mental State Examination score, and total gray matter volume, we identified significant increases in local gray matter volume in the left dorsolateral prefrontal cortex and left fusiform gyrus in the DS group. Moreover, rumination was found to partially mediate the relationship between the gray matter volumes in both regions and depressive symptoms. These findings indicated that the volumes of the left dorsolateral prefrontal cortex and left fusiform gyrus serve as potential neurostructural markers for subthreshold depression in non-clinical older female adults. Additionally, an underlying mediation mechanism in which gray matter volume affected depressive symptoms through individual rumination tendencies was proposed.

